# Suppressive Effect of *Lactococcus lactis* subsp. *cremoris* YRC3780 on a Murine Model of Japanese Cedar Pollinosis

**DOI:** 10.3390/pathogens11111347

**Published:** 2022-11-14

**Authors:** Kenji Uchida, Kenichi Iida, Ikumi Fujioka, Satoshi Hachimura, Osamu Kaminuma

**Affiliations:** 1 R&D Center, Yotsuba Milk Products Co., Ltd., Sapporo 061-1264, Japan; 2 Research Center for Food Safety, Graduate School of Agricultural and Life Sciences, The University of Tokyo, Tokyo 113-8657, Japan; 3 Department of Disease Model, Research Institute for Radiation Biology and Medicine, Hiroshima University, Hiroshima 734-8553, Japan

**Keywords:** allergic rhinitis, eosinophil, gastrointestinal microbiota, immunoglobulin E, Japanese cedar pollinosis, *Lactococcus lactis* subsp. *cremoris* YRC3780, nasal hyperresponsiveness

## Abstract

Accumulating evidence suggests that *Lactococcus lactis* subsp. *cremoris* YRC3780 isolated from kefir has the potential to alleviate allergic responses. Herein, we investigated the effect of YRC3780 on a murine model of Japanese cedar pollinosis (JCP). BALB/c mice immunized with cedar pollen extract (CPE) exhibited an increase in serum immunoglobulin E and developed nasal inflammatory responses including sneezing, nasal hyperresponsiveness, and nasal eosinophil accumulation upon intranasal allergen challenge. These responses were suppressed by the oral administration of YRC3780, although the effects on CPE-induced sneezing response and eosinophil infiltration were not statistically significant. Total fecal microbiota diversity was not affected by allergen immunization and challenge or by YRC3780 administration. However, the abundances of *Bifidobacteriales*, *Veillonellaceae*, *Lactococcus*, and *Lactococcus lactis* were larger and that of *Bacteroides* was smaller in YRC3780-treated mice compared with those in CPE-challenged and YRC3780-untreated mice. Our findings suggest the usefulness of YRC3780 for alleviating JCP.

## 1. Introduction

In recent years, the number of people with allergic diseases has been increasing annually worldwide. Specifically in Japan, the prevalence of allergic rhinitis (AR) as of 2019 was 49.2%, representing an increase of about 10% in 10 years [1]. Approximately 80% of AR patients in Japan (38.8% of the total population) suffer from Japanese cedar pollinosis (JCP) [1]. The current treatment of AR including JCP consists mainly of medications that relieve symptoms such as sneezing, rhinorrhea, and nasal congestion, and downregulate nasal inflammation. Subcutaneous and recently approved sublingual immunotherapies have the potential to modulate disease activity. However, prescribing medicines for the multitude of patients with AR raises concerns about substantial costs and morbidity from serious side effects [2]. Inspired by the successful oral application of bioproducts, particularly in sublingual immunotherapy, we became interested in the potential of food products that exhibit immunomodulatory effects to prevent the development and alleviate the symptoms of AR. Recent reports suggest that in addition to Benifuuki tea [3] and fermented fruit juice beverages [4], several foods containing lactic and acetic acid bacteria and bifidobacteria display efficacy in AR including JCP [5,6,7,8,9,10,11,12,13,14,15]. For example, based on double-blind placebo-controlled trials for JCP, *Lactobacillus paracasei* KW3110, *Lactobacillus acidophilus* L-55 and L-92, *Lactobacillus casei* Shirota, and *Bifidobacterium longum* BB536 have been reported to improve the patients’ symptoms to some degree [5,6,7,8,9,10].

*Lactococcus lactis* subsp. *cremoris* YRC3780 (hereafter YRC3780) is isolated from kefir, a traditional fermented milk product habitually consumed in the Caucasus region. YRC3780 has exhibited immunomodulatory effects in several animal studies, even in its heat-killed form. For example, in a bowel cancer-implanted mouse model, the serum IL-2 level and natural killer cell activity increased after oral live YRC3780 administration [16]. The allergen-induced production of IL-4 was suppressed, and IL-12 production was enhanced by co-culture of immunized mouse splenocytes with YRC3780, suggesting a beneficial effect on the Th1/Th2 balance. In an atopic dermatitis-like murine model of skin inflammation, oral heat-killed YRC3780 administration alleviated allergen-induced dermal responses and decreased the expression of IL-4 and IL-33 in CD4^+^ T cells in the Peyer’s patches and draining lymph nodes, respectively [17]. The effectiveness of live YRC3780 in human allergy as well has been suggested in several clinical studies [18,19]. In those rodent and human studies, no harmful effect of YRC3780 was reported.

**Figure 1:**
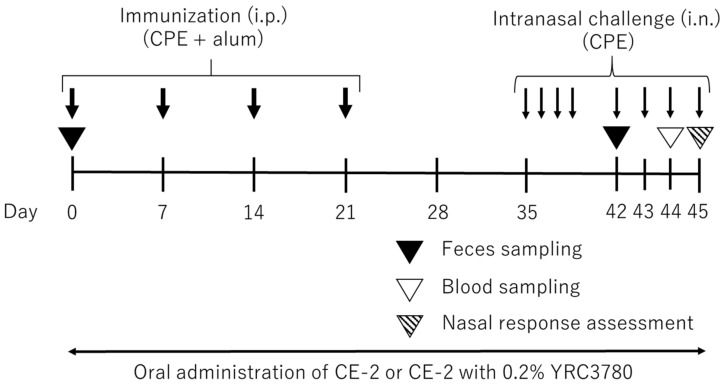
Experimental schedule for the mouse model of Japanese cedar pollinosis. BALB/c mice immunized by intraperitoneal (i.p.) injection of cedar pollen extract (CPE) plus alum were challenged by intranasal (i.n.) allergen injection. Mice were fed with standard rodent diet, CE-2 (Crea-Japan Inc., Tokyo, Japan), supplemented with or without *Lactococcus lactis* subsp. cremoris YRC3780 (YRC3780) throughout the experimental period. Blood and feces sampling and nasal response assessment were performed on the indicated days. The detailed experimental procedure is described in the Section 2.

In this study, we used an experimental model of JCP [20] in which mice are immunized and challenged with cedar pollen extract (CPE) to evaluate the efficacy of YRC3780. This murine JCP model (Figure 1) exhibits allergic responses similar to those in patients with JCP, i.e., elevation of the serum immunoglobulin E (IgE) level, CPE-induced sneezing response, eosinophil accumulation in the nasal mucosa, and nasal hyperresponsiveness (NHR), a pathophysiological feature by which AR symptoms are augmented [21]. We previously demonstrated the efficacy of dexamethasone and sublingual immunotherapy in the similar allergen-immunized and -challenged mouse models [22,23]. The effect of heat-killed YRC3780 on these allergen-induced responses was investigated by monitoring fluctuations in fecal microbiota.

## 2. Materials and Methods

### 2.1. Preparation of YRC3780

YRC3780 was isolated by Yotsuba Milk Products Co., Ltd. (Hokkaido, Japan), inoculated into M17 broth (Merck KGaA, Darmstadt, Germany), and cultivated for 16 h at 30 °C. We confirmed in a separate experiment that the resulting YRC3780 showed approximately 3–4 × 10^11^ colony-forming units/g. Then the cells were harvested by 8000 rpm centrifugation at 4 °C for 10 min, washed twice, and resuspended in sterilized distilled water. Following the heat treatment at 100 °C for 10 min, the cells were lyophilized and mixed with the standard rodent diet (CE-2, Crea-Japan Inc., Tokyo, Japan) at the concentration of 0.2% (*w/w*).

### 2.2. Administration of YRC3780 to Mice

Seven-week-old specific-pathogen-free female BALB/c mice were purchased from CLEA-Japan Inc. The mice received Milli-Q water and CE-2 under controlled conditions of temperature (25 ± 2 °C), humidity (50 ± 10%), and light (12 h light–dark cycle). After the habituation period of at least 7 days, the YRC3780 group received CE-2 containing 0.2% YRC3780 throughout the experimental period (Figure 1). Due to the requirement of the stable quality YRC3780 in CE-2 for 45-day consecutive administration, we used its heat-killed form in this study.

### 2.3. Mouse JCP Model

The experimental murine model of JCP was developed as previously described [20] with minor modifications (Figure 1). In brief, BALB/c mice were immunized with an intraperitoneal injection of 10 μg CPE (Cedar Pollen Extract-cj, Cosmo bio, Tokyo, Japan) emulsified with 0.5 mg alum (Imject Alum; Thermo Fisher Scientific Inc., Chicago, IL, USA) on days 0, 7, 14, and 21 (Figure 1). On days 35–38 and 42–45, the mice were challenged once daily with the two-time intranasal administration of 5.75 μL per nostril of 0.1 mg/mL CPE solution with a 5 min interval. The Normal group was prepared for negative control with the intraperitoneal and intranasal injection of saline in the same manner as CPE immunization and challenge (Figure 1).

Immediately after the last allergen challenge, nasal symptoms were evaluated by counting the number of sneezes for 5 min. After 6 h, nasal hyperresponsiveness (NHR) was evaluated by counting the number of sneezes for 5 min following the intranasal administration of 5 μL per nostril of 100 mM histamine (Nacalai Tesque Inc., Kyoto, Japan) solution. Immediately after the NHR assessment, nasal lavage was performed as described previously [23]. The number of leukocytes in the nasal lavage fluid (NALF) was counted using a hemocytometer. Through the microscopic examination of the cytocentrifuged preparation stained with Diff-Quik (Sysmex, Kobe, Japan), differential cell classification based on morphologic criteria was performed for at least 200 cells.

### 2.4. Measurement of Serum IgE Level

The allergen-induced IgE response was evaluated by measuring the total serum IgE. In order to reduce the invasive influence on the mice, peripheral blood collection was performed on day 44, which was close to, but different from, the days for nasal response assessment and feces sampling. The serum IgE level was assessed using an LBIS Mouse IgE ELISA Kit (Fujifilm Wako Pure Chemical, Osaka, Japan) according to the manufacturer’s instructions.

### 2.5. Microbiota Analysis

Metagenomic next-generation sequencing analysis was performed for investigating fecal microbiota. Feces collected from each mouse on days 0 and 42 was applied for DNA extraction using a ZymoBIOMICS DNA Miniprep Kit (ZYMO Research, Irvine, CA, USA) according to the manufacturer’s instructions. The V4 region of 16S rDNA was amplified with KAPA Hifi HS ReadyMix (NIPPON Genetics, Tokyo, Japan), F515 primer (5′-TCGTCGGCAGCGTCAGATGTGTATAAGAGACAGGTGYCAGCMGCCGCGGTAA-3′), and R806 primer (5′-TCGTCGGCAGCGTCAGATGTGTATAAGACAGGTGYCAGCMGCCGCGGTAA-3′), and then purified using AMPure XP (Beckman Coulter, Brea, CA, USA). The DNA library was prepared using a Nextera XT DNA Library Preparation Kit (Illumina Inc., San Diego, CA, USA), and then analyzed on iSeq100 (Illumina Inc.). The resulting FastQ sequence was applied for the OTU and quantitative insights into microbial ecology analyses in FASMAC (Kanagawa, Japan). The taxonomic classification was performed based on the Greengenes database provided by Lawrence Berkeley National Laboratory (Berkeley, CA, USA) and the SILVA database (SILVA131) provided by the Max Planck Institute for Marine Microbiology and Jacobs University (Bremen, Germany). Changes in the diversity of the microbiota at day 42 from the start of the experiment were evaluated by assessing the OTU number, Chao1 index, and Shannon diversity index, and the composition of several bacterial species.

### 2.6. Statistical Analysis

The data are presented as the mean ± standard error of the mean. The difference among three groups was analyzed by using the Kruskal–Wallis test with Dunnet’s multiple comparison tests in BellCurve for Excel (Social Survey Research Information Co., Tokyo, Japan). A *p*-value less than 0.05 was considered to indicate statistical significance.

## 3. Results

### 3.1. Effect of YRC3780 on Serum IgE Response

**Figure 2:**
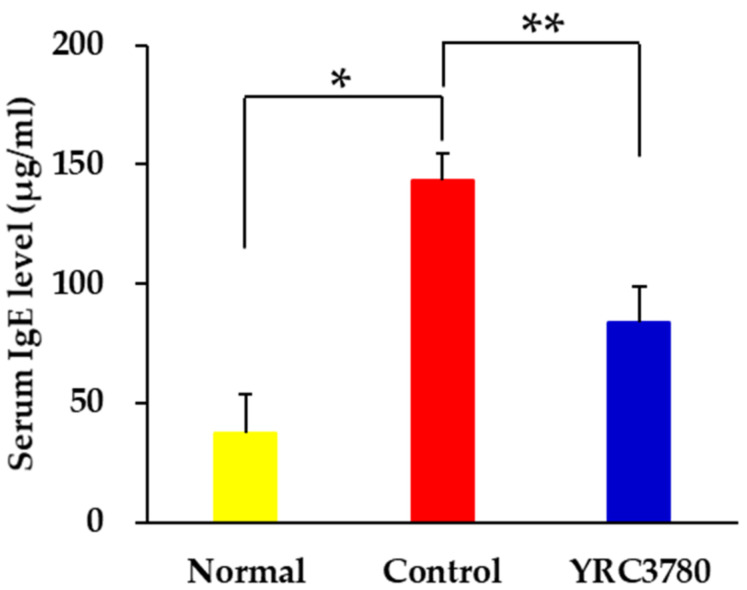
Effect of YRC3780 on the serum immunoglobulin E (IgE) response. Cedar pollen extract-immunized mice were challenged by intranasal allergen injection with (YRC3780) or without (Control) oral administration of YRC3780. Non-immunized and -challenged mice (Normal) were the negative control. The serum IgE level was evaluated on day 44, as shown in Figure 1. Data are expressed as means ± standard error of the mean of 12 mice in each group. * *p* < 0.05, ** *p* < 0.01.

The murine JCP model (Figure 1) was established by immunization and challenge with CPE as described previously [20]. The administration of YRC3780 was achieved by the free oral intake of diet containing 0.2% YRC3780. The allergen-induced IgE response was evaluated by assessing the serum IgE level in the peripheral blood. The serum IgE level was significantly elevated in allergen-immunized and -challenged mice (Control group) in comparison with non-immunized and -challenged mice (Normal group). The allergen-induced serum IgE response was significantly suppressed in YRC3780-treated mice (YRC3780 group) (Figure 2).

### 3.2. Effect on Nasal Responses

**Figure 3:**
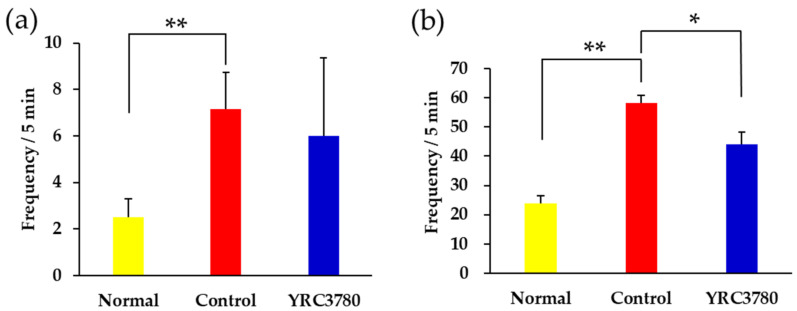
Effect of YRC3780 on allergen-induced sneezing and nasal hyperresponsiveness (NHR). Cedar pollen extract-immunized mice were challenged by intranasal allergen injection with (YRC3780) or without (Control) oral administration of YRC3780. Non-immunized and -challenged mice (Normal) were the negative control. The allergen-induced sneezing response was evaluated by counting the frequency of sneezes immediately after the last allergen challenge (**a**). Allergen-induced NHR was evaluated by counting the frequency of histamine-induced sneezes 6 h after the last allergen challenge (**b**). Data are expressed as means ± standard error of the mean of 12 mice in each group. * *p* < 0.05, ** *p* < 0.01.

The effect of YRC3780 on allergen-induced nasal responses was investigated. Intranasal CPE administration evoked a significant increase in the frequency of sneezes in the Control group. The allergen-induced sneezing response was slightly, but not significantly, alleviated by YRC3780 administration (Figure 3a, *p* = 0.08). Our previous study demonstrated that the allergen-induced sneezing response in this model represents T cell-dependent nasal NHR rather than IgE-dependent mast cell degranulation [24]. Therefore, the effect of YRC3780 on allergen-induced NHR was examined. The histamine-evoked sneezing response was significantly augmented in the Control group, indicating the development of NHR. The NHR response was significantly suppressed by YRC3780 administration (Figure 3b).

### 3.3. Effect on Eosinophil Accumulation in the Nasal Mucosa

**Figure 4:**
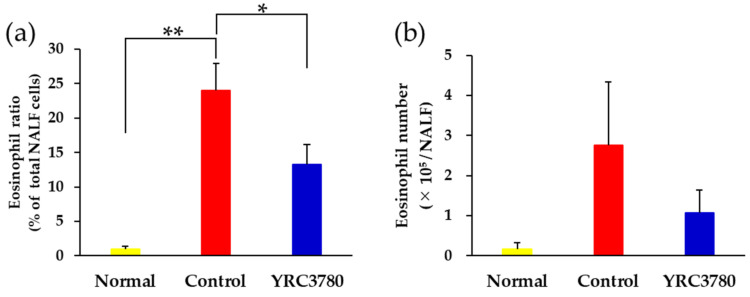
Effect of YRC3780 on allergen-induced nasal eosinophil accumulation. Cedar pollen extract-immunized mice were challenged by intranasal allergen injection with (YRC3780) or without (Control) oral administration of YRC3780. Non-immunized and -challenged mice (Normal) were the negative control. The ratio (**a**) and number (**b**) of eosinophils in the nasal lavage fluid (NALF) was evaluated 6 h after the last allergen challenge. Data are expressed as means ± standard error of the mean of 11 (Control) or 12 (YRC3780, Normal) mice. * *p* < 0.05, ** *p* < 0.01.

The allergen-induced accumulation of eosinophils in the nasal mucosa was evaluated by the ratio of eosinophils to total inflammatory cells and the number of eosinophils recovered in nasal lavage fluid (NALF). CPE challenge to immunized mice evoked a significant increase in the ratio of eosinophils in NALF. The allergen-induced increase in the NALF eosinophil ratio was suppressed by YRC3780 administration (Figure 4a). Essentially the same, but not significant, tendency was also observed in the number of NALF eosinophils.

### 3.4. Influence on Fecal Microbiota

#### 3.4.1. Diversity of Microbiota

**Figure 5:**
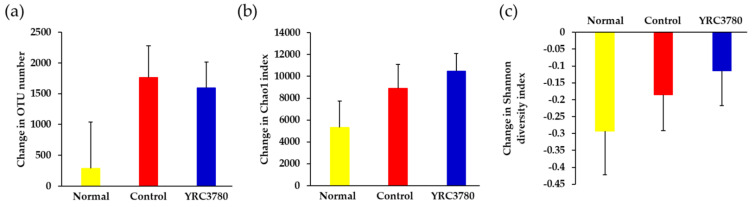
Effects of YRC3780 on fecal microbiota diversity. Cedar pollen extract-immunized mice were challenged by intranasal allergen injection with (YRC3780) or without (Control) oral administration of YRC3780. Non-immunized and -challenged mice (Normal) were the negative control. The microbiota composition in feces was analyzed on days 0 and 42, as indicated in Figure 1. Changes in the operational taxonomic unit (OTU) number (**a**), Chao1 index (**b**), and Shannon diversity index (**c**) from the start of the experiment were calculated. Data are expressed as mean ± standard error of the mean of 12 mice in each group.

The change in the diversity of fecal microbiota during the experimental period was examined, as shown in Figure 1. The basal levels of operational taxonomic unit (OTU) number (3914 ± 608) and indicators of α-diversity such as Chao1 index (15678 ± 1615) and Shannon diversity index (4.21 ± 0.10) were comparable to those demonstrated in previous studies [25]. A slight increase in the OTU number was observed in the Normal group at day 42 from the start of the experiment. CPE immunization and challenge tended to upregulate the OTU number, but not significantly, regardless of YRC3780 administration (Figure 5a). Probably due to the spontaneous fluctuation of the OTU number, a slight increment in the Chao1 index, and conversely, a reduction in the Shannon diversity index were observed in the Normal group, although these parameters were not affected by allergen or YRC3780 treatment (Figure 5b,c). Since these differences were not statistically significant, the negligible contribution of YRC3780, if any, to the total diversity of gastrointestinal microbiota was suggested.

#### 3.4.2. Bacterial Species

**Figure 6:**
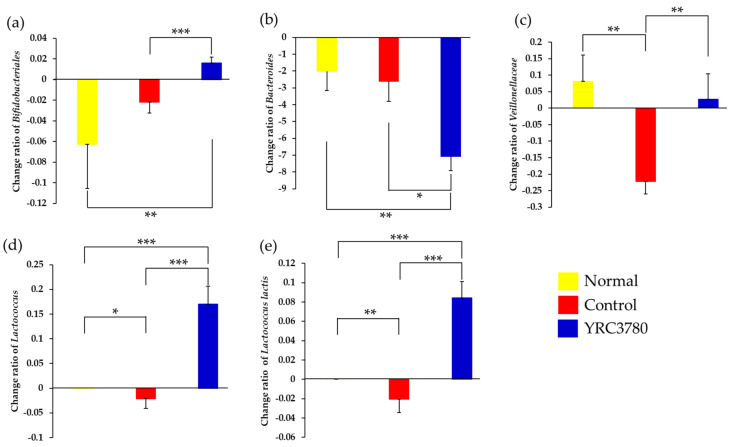
Effect of YRC3780 on changes in fecal bacterial species. Cedar pollen extract-immunized mice were challenged by intranasal allergen injection with (YRC3780) or without (Control) oral administration of YRC3780. Non-immunized and -challenged mice (Normal) were the negative control. The microbiota composition in feces was analyzed on days 0 and 42, as indicated in Figure 1. The change ratios in the abundance of *Bifidobacteriales* (**a**), *Bacteroides* (**b**), *Veillonellaceae* (**c**), *Lactococcus* (**d**), and *Lactococcus lactis* (**e**) from the start of experiment were calculated. Data are expressed as mean ± standard error of the mean of 12 mice in each group. * *p* < 0.05, ** *p* < 0.01, *** *p* < 0.001.

The influence of oral YRC3780 administration on several fecal bacterial species was evaluated by analyzing the change in microbiota composition during the experimental period. Spontaneous decreases in *Bifidobacteriales* and *Bacteroides* and an increase in *Veillonellaceae* were observed even in the Normal group at day 42 from the start of the experiment (Figure 6). Immunization and challenge with CPE significantly decreased the composition of *Veillonellaceae*, *Lactococcus*, and *Lactococcus lactis*. In the Control group, the composition of *Bifidobacteriales*, *Veillonellaceae*, *Lactococcus*, and *Lactococcus lactis* was significantly higher and that of *Bacteroides* was lower in the YRC3780 group. Even when compared with the Normal group, the YRC3780 group showed significantly higher abundances of *Bifidobacteriales*, *Lactococcus*, and *Lactococcus lactis* and lower levels of *Bacteroides* (Figure 6), suggesting the existence of the allergen-independent effect of YRC3780. In contrast, the CPE-mediated downmodulation of *Veillonellaceae* composition recovered with YRC3780 administration; this outcome was in parallel with the effect of YRC3780 on allergen-induced serum IgE, NHR, and nasal eosinophil responses.

## 4. Discussion

The present study demonstrated that YRC3780 has the potential to alter the pathogenesis of AR by downregulating IgE production, NHR development, and nasal inflammation with decreasing eosinophil ratio. Our findings suggest a possible relationship between these beneficial effects of YRC3780 and its influence on several gastrointestinal bacterial species, despite a lack of effect on total microbiota diversity.

The downmodulating effect of YRC3780 on IgE synthesis is supported by our previous study demonstrating decreased IL-4 expression in splenocytes in vitro and in Peyer’s patch CD4^+^ T cells in vivo with YRC3780 treatment [17]. Since the mice were immunized with CPE, the total IgE response was expected to reflect a CPE-specific IgE response. Therefore, this effect is probably related to the tendency toward suppression of the allergen-induced sneezing response principally caused by IgE-mediated mast cell degranulation. As further proof of concept, we recently demonstrated the suppression of IgE- and mast cell-dependent itchy eyes in adult patients with AR in a randomized, double-blind, placebo-controlled, 8-week trial of drinkable yogurt containing YRC3780 [19]. A decreased allergen-induced serum IgE response in CPE-immunized mice that received a *Lactobacillus helveticus* strain has also been reported [26].

The allergen-induced sneezing response is related to NHR. In our murine AR model, the development of NHR was largely dependent on allergen-specific T cells. The augmentation of the histamine-induced sneezing response in allergen-immunized and -challenged mice, as shown in this study, was suppressed by depleting CD4^+^ T cells. Furthermore, mice transplanted with in vitro-differentiated T cell subsets developed significant NHR upon allergen challenge independent of IgE- and mast cell-related responses [24]. These observations indicate that CD4^+^ T cells are likely one of the primary targets of YRC3780-mediated NHR suppression. In addition to the ability of YRC3780 to suppress IL-4 activity, we recently demonstrated that YRC3780 augments the production of IFN-γ in the co-culture of antigen-presenting cells and CD4^+^ T cells. In this regard, YRC3780 potentially improves the Th1/Th2 balance. A similar effect has been reported in studies of other lactic acid bacteria. In a murine model of birch pollen allergy, Repa et al. demonstrated the upregulation of IgG2a and IFN-γ production by *Lactococcus lactis* and *Lactobacillus plantarum* administration [27]. The downregulation of allergen-induced peritoneal eosinophilia and increased serum IgG2a/IgG1 ratio in CPE-immunized mice by administrating a *Lactobacillus gasseri* strain has also been reported [28].

Allergen-induced local eosinophil accumulation is also mediated by CD4^+^ T cells. We demonstrated in an earlier study [29] that the airway eosinophilia in allergen-specific T cell-transferred mice was abrogated by anti-IL-5 neutralizing antibody treatment. In addition, administering a *Lactobacillus paracasei* strain to perinatal maternal mice decreased birch pollen-induced bronchial inflammation with eosinophil accumulation and IL-5 production in offspring [30]. We need to confirm the effect of YRC3780 on IL-5 synthesis. Wang et al. demonstrated the reduced expression of IL-33, another eosinophil-activating cytokine, in the draining lymph nodes of atopic dermatitis model mice treated with YRC3780 [17]. In addition to T cell-derived factors responsible for NHR development, the mechanisms underlying the effect of YRC3780 on CD4^+^ T cells remain to be explored.

The modification of gastrointestinal microbiota by several lactic acid bacteria, even in their heat-killed form, as in this study, has been reported [31,32]. In addition to live microbe-based probiotics, the concept of postbiotics has recently attracted attention for maintaining gut conditions. Postbiotics based on non-viable microorganisms and bacterial-free extracts, as we used in this study, provide a benefit to the host by enhancing the bioactivities of probiotics [33]. Although we did not observe an effect on total microbiota diversity, the composition of several bacterial strains was affected by YRC3780 administration. In particular, the recovery of *Veillonellaceae* from allergen-induced downregulation is linked to YRC3780′s effect on AR responses. A direct effect on allergic responses has not been reported; however, a strong association of gastrointestinal *Veillonellaceae* content with food sensitization conditions [34] and atopy [35] has been reported. Lynch et al. demonstrated the correlation of bronchial asthma development with the presence of *Veillonellaceae* in environment house dust [36]. The direct effect of *Veillonellaceae* on allergic responses deserves further examination.

In conclusion, based on the suppressive effect on allergen-induced serum IgE production, NHR, and nasal eosinophilia, the oral intake of YRC3780, even in its heat-killed form, is a promising addition to the currently available medication for AR including CPE-mediated JCP. Additional research is needed to further delineate the usefulness of YRC3780, and its underlying mechanisms, including gastrointestinal microbiota modification.

## Data Availability

Not applicable.
